# Adherence to General Medical Council guidance regarding disclosure of alternative treatments during the consent process

**DOI:** 10.1308/rcsann.2024.0016

**Published:** 2024-05-15

**Authors:** GS Bethell, RA Wheeler, NJ Hall

**Affiliations:** ^1^ University of Southampton, UK; ^2^ University Hospital Southampton NHS Foundation Trust, UK

**Keywords:** Informed consent, Appendicitis, General Medical Council

## Abstract

**Introduction:**

General Medical Council (GMC) guidelines dictate that reasonable alternatives to treatment should be disclosed during the consent process. We aimed to determine whether GMC guidelines on disclosure of alternatives during consent are being followed in a real-world example which is disclosure of non-operative management as an alternative to appendicectomy in uncomplicated paediatric appendicitis.

**Methods:**

We undertook a retrospective single-centre observational study and national consultant specialist paediatric surgeon survey. Two groups of 50 consecutively treated children (<16 years) with acute uncomplicated appendicitis were included in the observational study during two periods. UK-based consultant surgeons who treat appendicitis were included in the national survey. The main outcomes were disclosure and use of non-operative management (NOM) as an alternative to appendicectomy.

**Results:**

Overall, in the observational study, NOM was disclosed in 30 (30%) children and 77% (23/30) opted for this treatment method when it was disclosed. There were 83 survey respondents representing all 25 eligible specialist paediatric surgery centres. Ten (12%) consultants reported routinely offering NOM, 39 (47%) offer it in select circumstances, and 34 (41%) never offer NOM. Only 25 (30%) respondents always disclose NOM as an alternative to appendicectomy, whereas 22 (27%) never do. Consultants who never disclose NOM are more likely to prefer appendicectomy over NOM compared with those who always disclose it (*p*<0.001).

**Conclusion:**

In this illustrative clinical scenario, observed and reported practice regarding disclosure of alternative treatments during the consent process do not meet GMC guidance. This risks depriving children and caregivers of a choice that they are entitled to.

## Introduction

Before any treatment, and more formally in surgical procedures, consent is required. General Medical Council (GMC) guidelines dictate that reasonable alternatives to any form of treatment should be disclosed by the clinician during the consent process, even if this is not part of their preferred practice.^1^ There are several examples of legal cases that ruled in favour of a claimant when an alternative treatment method was not disclosed and documented during the consent process. Probably the most widely known judgement is that of the Supreme Court.^2,3^ In this case Montgomery, a pregnant lady of small stature with diabetes, did not have an alternative to vaginal delivery disclosed by her obstetrician despite enquiring whether her baby’s size could be problematic during delivery. Unfortunately, the delivery was complicated owing to shoulder dystocia and the infant suffered a hypoxic insult with subsequent progression to cerebral palsy. Montgomery stated that had she had known this risk and been made away of the option of caesarean section, she could have opted for this alternative method of delivery.

Shared decision making has been described as “the pinnacle of patient-centred care” and is the process in which a patient, their family, and other members of the healthcare team decide on a healthcare plan together to ensure it aligns to a patient’s wishes and values.^4^ It is not possible to participate in shared decision making if alternative treatment options are not disclosed. Failure to disclose reasonable treatment options deprives patients and families of choice, which they are entitled to.

There has been growing interest and an increasing evidence base supporting non-operative management (NOM), as an alternative to appendicectomy, for uncomplicated paediatric appendicitis in recent years. NOM, consisting of antibiotic administration, analgesia and observation has been shown to be safe and effective in a number of prospective observational studies and randomised controlled trials (RCTs) in children.^5–10^ The reported success rate of NOM at 1 year is as high as 90% and there may be benefits over surgical treatment including a reduction in overall complication rate and faster return to daily activities.^8,10,11^

Given the growing evidence base supporting use of NOM as a safe and effective alternative to appendicectomy for uncomplicated appendicitis, it serves as a useful real-world example with which to determine whether the process of gaining consent for treatment of children with uncomplicated appendicitis meets the standard set by the GMC.^10,11^ We investigated this at the patient level in an observational study and at the national level via a survey of consultant specialist paediatric surgeons.

## Methods

### Observational study

Following local approval a retrospective case note and consent form review was undertaken for 2 groups of 50 consecutive children treated for presumed uncomplicated appendicitis at a single specialist paediatric surgical centre. The sample size was decided a priori to allow adequate comparison between groups while also being achievable within a single specialist centre setting. Appendicitis was presumed to be uncomplicated if the validated complicated appendicitis score was less than 4.^12^ The first group of children were treated from April 2018, immediately after a feasibility RCT of NOM of uncomplicated appendicitis at our hospital (group A), whereas cases in the second group were taken from November 2020, during recovery from the COVID-19 pandemic (group B).^10^ These time points were used so that intervention during the RCT, or access to operating theatres during the pandemic, did not directly impact practice. This part of the study was intentionally retrospective to ensure that knowledge of data collection did not impact clinical practice.

### National consultant survey

Consultant specialist paediatric surgeons who treat acute appendicitis were invited to complete a 21-item survey (see online supplementary material) electronically distributed using REDCap to all specialist paediatric surgical centres in December 2022.^13^ The survey was distributed to a lead consultant in each centre who then distributed this only to colleagues who treat this condition. Trainees and other non-consultants were not eligible to participate. If a centre was currently recruiting to an ongoing RCT (ISRCTN16720026) of NOM in uncomplicated appendicitis then practice and views were requested immediately before commencing recruitment for this. Views and practice were requested for presumed uncomplicated appendicitis only.

### Outcomes

Outcomes of interest in the observational study were disclosure and use of NOM determined by examination of case notes and consent forms. Outcomes of interest for the survey were reported practice regarding disclosure and offering of NOM along with practice regarding shared decision making. In addition, perceived effectives of NOM in uncomplicated appendicitis was invited. Free-text reasons for these responses were requested.

### Patient and public involvement

This research was co-designed and fully supported by our patient and public involvement group, which consists of parents and young people, the majority of whom have had appendicitis themselves or have had a child with the disease. Members of this group with lived experience reported that an alternative to appendicectomy was infrequently disclosed to them during consent and therefore felt that the research question is particularly important and relevant.

### Statistical analysis

Data are reported as number and percentage with comparison between time points using Fisher’s exact test. Preferred treatment strategy of survey respondents was collected on a scale of 0, indicating preference for appendicectomy, to 100, which indicated preference for NOM. This score was compared between disclosure of NOM practice using the Kruskal–Wallis test. Statistical analyses were undertaken using StataSE v17 (StataCorp LLC, College Station, TX, USA). A *p* value of <0.05 was considered as statistically significant.

## Results

### Observational study

Of the 100 included children, 61 were male with a median age of 10.5 (range 2–15) years. NOM was disclosed overall in 30 (30%) children and NOM was disclosed more frequently in group B (November 2020 onwards) than A (April 2018 onwards) (27 [54%] vs 3 [6%] children; *p*<0.0001). When NOM was disclosed 23/30 (77%) children and/or their caregivers opted for this treatment method and again this was more frequent in group B than A (21 [42%] vs 2 [4%] children; *p*<0.0001).

### National consultant survey

**Figure 1:**
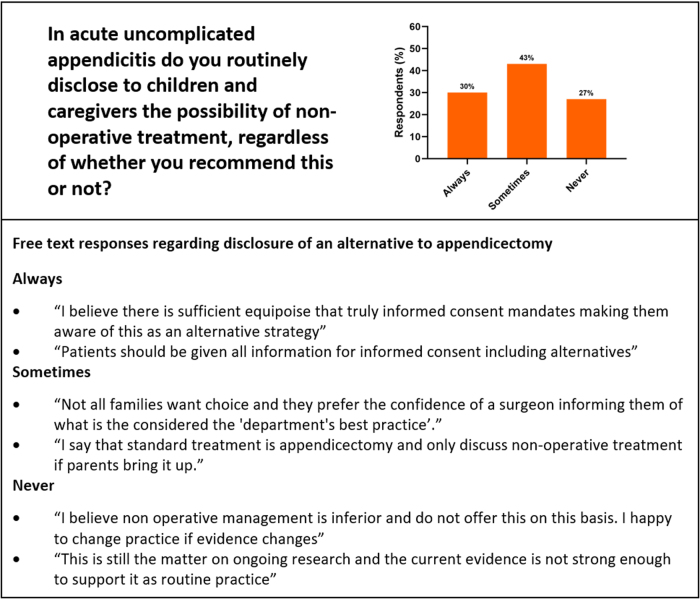
Respondents' reported practice regarding disclosure of an alternative to appendicectomy with a selection of free-text explanations for their response

**Figure 2:**
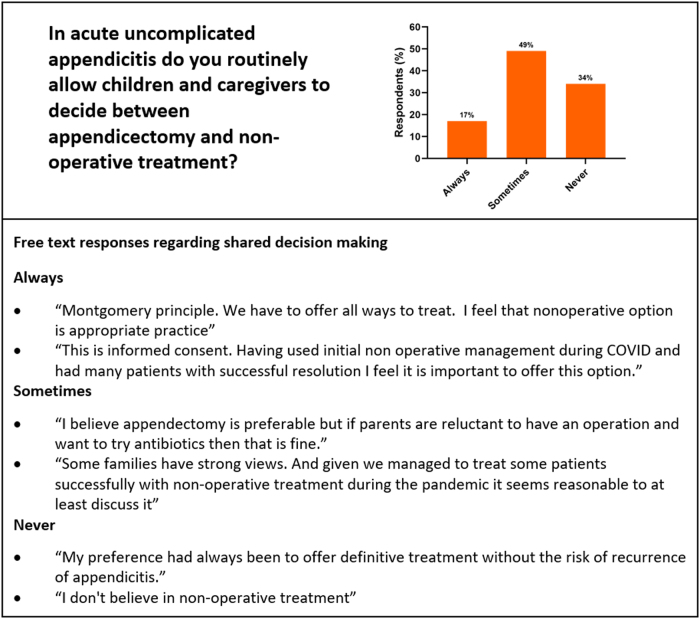
Respondents' reported practice regarding shared decision making with children and caregivers with a selection of free-text explanations for their response

There were 83 respondents with representation from all 25 UK specialist paediatric surgery centres that treat acute appendicitis (*n*=25/25, 100%). When asked about current practice, 10 (12%) consultants reported routinely offering NOM, 39 (47%) do in select circumstances and 34 (41%) respondents never offer NOM. No respondents indicated that they never offer appendicectomy. Only 25 (30%) respondents always disclose NOM as an alternative to appendicectomy, whereas 22 (27%) report that they never do (Figure 1). Regarding shared decision making, 14 (17%) consultants always allow children and caregivers to decide on treatment strategy in uncomplicated appendicitis, whereas 28 (34%) never do (Figure 2).

**Figure 3:**
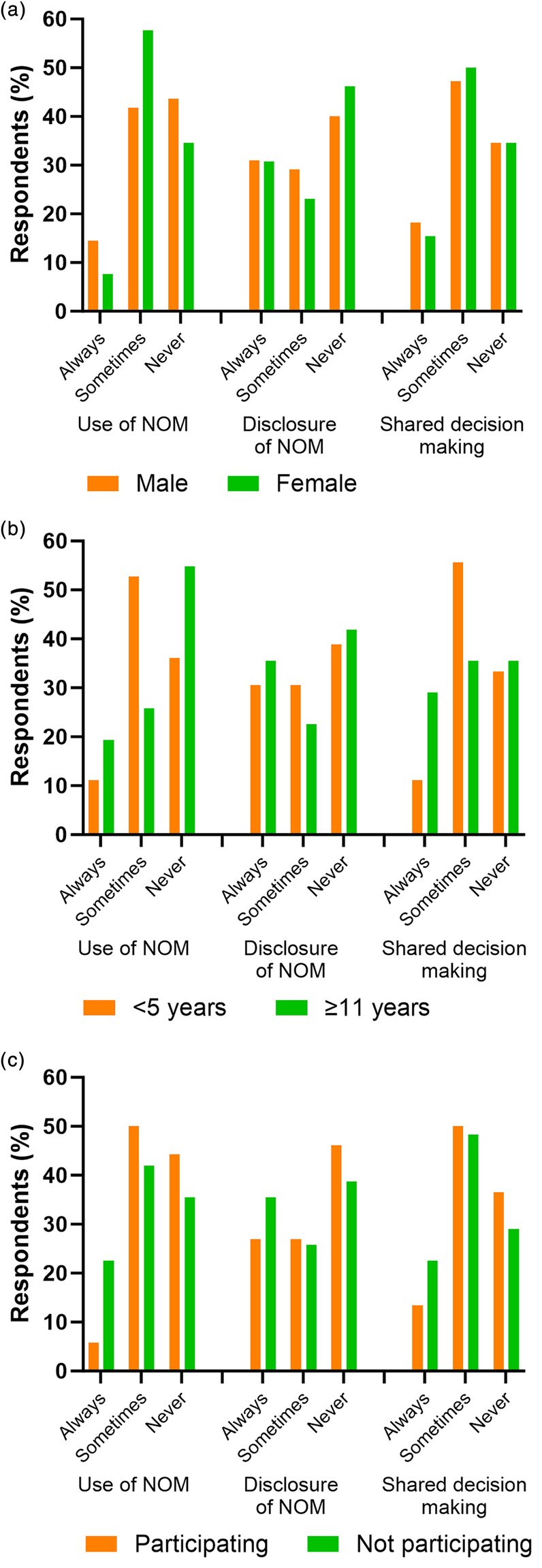
Use of, disclosure of and shared decision making regarding non-operative management (NOM) comparing (a) male vs female respondents, (b) least vs most consultant experience and (c) participation v non-participation in a randomised controlled trial of NOM in paediatric uncomplicated appendicitis

Current practice (*p*=0.37), practice regarding disclosure (*p*=0.82) and use of shared decision making (*p*=0.95) were also similar between male (*n*=55) and female (*n*=26) consultants, where sex was disclosed (Figure 3a). Consultant experience was less than 5 years in 36 (43%) respondents, 6–10 years in 16 (19%) and 11 years or more in 31 (37%). Reported current practice (*p*=0.08), practice regarding disclosure (*p*=0.76) and use of shared decision making (*p*=0.12) were similar between those with the most vs least consultant experience (Figure 3b). Of the survey respondents, 52/83 (63%) consultants were from centres where recruitment to a RCT of NOM in uncomplicated paediatric appendicitis was ongoing. Despite this, current practice (*p*=0.08), practice regarding disclosure (*p*=0.69) and use of shared decision making (*p*=0.52) were similar between those that were and were not recruiting to the RCT (Figure 3c).

**Figure 4:**
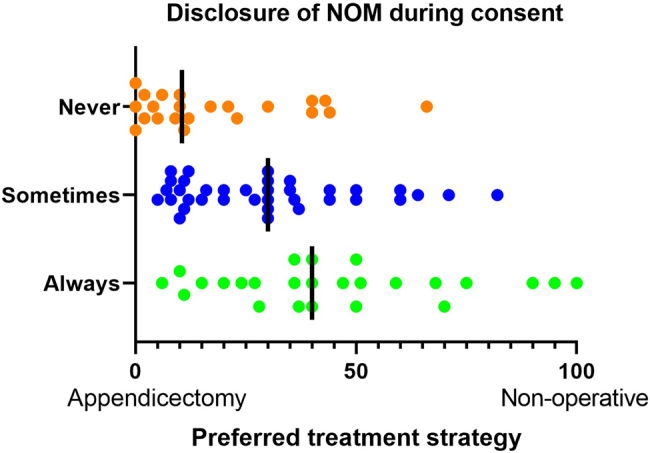
Preferred treatment strategy of survey respondents stratified by their practice regarding disclosure of an alternative to appendicectomy (*p*<0.001). Each symbol represents an individual response and vertical lines are medians

Consultants who never disclose NOM as an alternative to appendicectomy were more likely to prefer appendicectomy to NOM compared with those that sometimes, and those that always, disclose it (*p*<0.001) (Figure 4). A range of opinions were elicited in the free-text responses providing evidence that there is an understanding of GMC guidance and the Montgomery principle, nevertheless there is an unwillingness to implement NOM in current practice.

## Discussion

This study found that disclosure of NOM as an alternative to appendicectomy occurs infrequently in uncomplicated paediatric appendicitis. According to our data, current practice, observed in a specialist centre and reported nationally, does not meet GMC guidance, which states that valid treatment alternatives should be discussed during the consent process. These results suggest that children and their caregivers are being deprived of the opportunity to contribute to shared decision making. Furthermore, surgeons may not be obtaining consent of a fully informed nature in this common clinical scenario.

Despite observed disclosure of NOM as an alternative to appendicectomy in only 30% of children and only 30% of consultants reporting they consistently offer this modality, there are some positive findings to this study. Disclosure of NOM in the observational study has increased dramatically from the initial study period in 2018 to the subsequent period beginning in 2020. There are several possible reasons for this. First, the results of a UK-based feasibility RCT were made available between these time points, which no doubt raised awareness of this treatment modality and provided experience of using this.^10^ Second, the COVID-19 pandemic caused a rapid change in practice with increased use of NOM in appendicitis following guidance from the Royal College of Surgeons of England.^11,14,15^ This provided surgeons with experience of using NOM as a treatment modality even if it was not their preferred choice of treatment. It is also reassuring to see that many surgeons recognise the importance of fully informed consent in the survey, as evidenced by the free-text responses. Finally, a number of surgeons were found to strongly prefer appendicectomy as a treatment strategy, rather than NOM, yet indicated that they always disclose NOM during the consent process. This practice clearly meets GMC guidance and gives children and caregivers an opportunity to request this treatment method even if the consultant taking consent does not recommend, or even offer, this themselves.

Contrary to these positive points, there were some concerning practices identified and reported, particularly in the free-text responses. Many free-text responses from consultants that never disclose NOM or allow shared decision making were based around the perceived efficacy of NOM. The Getting It Right First Time abdominal pain pathway provides a national way of managing children with abdominal pain and specifically addresses this point.^16^ It specifies that, given the emerging evidence regarding NOM in uncomplicated appendicitis, this should be discussed as part of the consent process with reference to the Montgomery principle. It states this regardless of preferred treatment approach of the surgeon.

A recent judgement confirms that the correct legal test for determining the full extent of the reasonable treatment options available (in this instance, for uncomplicated appendicitis in children) remains whether the practice of the doctor which is in issue is supported by a reasonable or responsible body of professional opinion.^17^ By contrast, the decision as to which of the available options to choose remains a matter for the patient, not the doctor, so if needs be will ultimately be determined by a court, and not by expert medical opinion.^3^ It is self-evident that on this basis, NOM is supported by a reasonable and responsible body of paediatric surgeons. It is notable, if alarming, that during a 13-year data collection of clinical–legal enquiries from children’s clinicians, although 30 enquiries touched upon the provision of reasonable disclosure prior to consent, only one enquiry related to the substance of disclosure; that did relate to alternative options for the treatment of appendicitis, but hardly reflects an active debate in the field of surgical disclosure prior to seeking consent. That debate is overdue.^18^

There are also clinical–legal implications of not providing fully informed consent to children and caregivers. Appendicitis is known to be a common disease where litigation occurs in paediatric surgery and, in fact, a study from the United States found that appendicitis was the most common condition where litigation occurred.^19^ Moreover, substandard informed consent was the reason cited for around 5% of these legal claims. If NOM is discussed as an alternative treatment method, where appropriate, and documented, then the benefit of this disclosure might extend beyond the child and caregiver by preventing litigation from failure to provide reasonable information. In addition, a systematic review with the aim of determining whether shared decision making can reduce malpractice mitigation found evidence that ignoring or failing to understand patient preferences puts clinicians at higher risk of litigation.^20^

### Study limitations

This study is limited by reporting actual practice at only a single centre, but the national survey allows a perspective of all specialist paediatric surgery centres. This report focuses on disclosure during consent for a single condition and does not report other data such as efficacy of NOM in uncomplicated appendicitis; however, several other studies have achieved this.^5,8–11^ It may be that NOM as an alternative treatment was disclosed in the observational study and not documented; however, a medicolegal review would conclude that if a conversation was not documented then it did not occur. Standardised paper or electronic consent forms were not used during the study period in our centre; however, they could have increased disclosure of alternative treatment options if their use was agreed by all consultants. In addition, uncomplicated, rather than complicated, appendicitis was diagnosed retrospectively using a validated score based on case note review rather than being documented by the clinician at the time of taking consent. Although it is theoretically possible that a case was not suitable for NOM at the time, in reality this is unlikely given the diagnostic test parameters of the score, which has a very low false-negative rate. Hence, it is unlikely that a surgeon would have deemed someone to have complicated disease and not offered NOM for this reason. Finally, we only distributed the survey to consultant surgeons and it is likely that many discussions and consent processes are undertaken by trainees for appendicitis. Although we cannot be certain that trainees’ practice is congruous with their consultant supervisors, we anticipate there is discussion and perhaps a policy in most centres regarding these treatment alternatives such that consultant views reflect actual practice.

## Conclusion

This work, using two methodologies, reports a lack of adherence to GMC guidelines regarding consent among specialist paediatric surgeons in the context of discussing treatments for uncomplicated appendicitis. Given recent case law regarding the threshold for discussing reasonable alternative treatments, we recommend that NOM should be disclosed as an alternative to appendicectomy in presumed uncomplicated appendicitis. More generally, these data act as a reminder to clinicians from all specialities that disclosure of alternatives to any form of treatment, where a reasonable alternative exists, should be made. Failure to do so risks undermining the provision of fully informed consent, may deprive patients and caregivers of a choice to which they are entitled and may expose clinicians to greater risk of litigation.

## Data availability statement

Anonymised data available following approval of study proposal to the corresponding author. These will be available from date of publication until 10 years has elapsed.

## Author contributions

GB, RW and NH: conceptualisation. GB: data curation. GB and NH: formal analysis. GB and NH: investigation. GB and NH: methodology. GB: project administration. NH: resources. NH: supervision. GB: visualisation. GB: writing – original draft. RW and NH: writing – review & editing.

## Funding

George Bethell is funded by the National Institute of Health Research Doctoral Fellowship programme (NIHR302541). The views expressed are those of the authors and not necessarily those of the NHS, the NIHR or the Department of Health.
